# The Association Between Obesity Indices and Non-Alcoholic Fatty Liver Disease in Patients with Ankylosing Spondylitis

**DOI:** 10.5152/ArchRheumatol.2025.25108

**Published:** 2025-12-01

**Authors:** Oznur Kutluk, Nimet Ezgi Dabak, Ayca Ayse Yucel, Ahmet Sukru Alparslan, Fatih Cay

**Affiliations:** 1 Department of Rheumatology, ntalya Research and Training Hospital,Antalya, Türkiye; 2 Department of Physical Medicine and Rehabilitation, Antalya Research and Training Hospital, Antalya, Türkiye; 3 Department of Radiology, Antalya Research and Training Hospital, Antalya, Türkiye

**Keywords:** Ankylosing spondylitis, non-alcoholic fatty liver disease, obesity

## Abstract

**Background/Aims:**

This study aimed to investigate the relationship between obesity indices and non-alcoholic fatty liver disease (NAFLD) in patients with ankylosing spondylitis (AS).

**Materials and Methods:**

A total of 170 patients with AS were included in this observational study with both prospective and retrospective components. Anthropometric measurements (height, weight, waist circumference [WC]) were prospectively recorded by the investigators during routine clinical visits. Obesity indices, including body mass index (BMI), WC, and waist-to-height ratio (WHtR), were calculated. Non-alcoholic fatty liver disease was diagnosed based on existing abdominal ultrasonography reports. Laboratory data, including liver enzymes, lipid profiles, and inflammatory markers, were retrospectively collected. The diagnostic performance of WC for NAFLD was assessed using receiver operating characteristic (ROC) curve analysis.

**Results:**

Non-alcoholic fatty liver disease was detected in 57% of AS patients. Higher BMI and WHtR were significantly associated with both the prevalence and severity of NAFLD (*P* < .001). Patients with NAFLD exhibited significantly higher levels of C-reactive protein, triglycerides, and alanine aminotransferase, as well as lower high-density lipoprotein levels, compared to those without NAFLD (*P* < .05). Additionally, the aspartate aminotransferase/alanine aminotransferase ratio was <1 in 66% of NAFLD patients, suggesting its potential as a biochemical marker for NAFLD in AS. The ROC analysis identified WC cutoff values of 100 cm for males and 90 cm for females, demonstrating high sensitivity and specificity in predicting NAFLD.

**Conclusion:**

Obesity is strongly associated with NAFLD in AS patients. Body mass index and WHtR may serve as valuable indicators for assessing hepatic steatosis risk. Routine metabolic evaluations, including obesity-related parameters and liver enzyme levels, could facilitate the early detection of NAFLD in AS patients. Further prospective studies are warranted to clarify the causal relationship between obesity and liver involvement in AS.

**Main Points**

- Non-alcoholic fatty liver disease (NAFLD) was quite frequent among patients with ankylosing spondylitis (AS).
- Waist circumference (WC), body mass index, and waist-to-height ratio (WHtR) were all related to the presence and severity of NAFLD.
- Patients with NAFLD had higher C-reactive protein, alanine aminotransferase, and triglyceride levels, and lower high-density lipoprotein levels compared to those without NAFLD.
- Receiver operating characteristic curve analysis showed that a WC of 100 cm for men and 90 cm for women could indicate a higher risk of NAFLD.
- An increased WHtR, as a simple indicator of abdominal obesity, was directly linked with liver fat accumulation and may be used in daily practice for NAFLD screening in AS patients.

## Introduction

Ankylosing spondylitis (AS) is a long-standing inflammatory condition that mainly involves the spine and sacroiliac joints. This condition, which typically emerges in young adults, may lead to axial skeletal deformities and movement restrictions in later stages.^1^ It is well established that AS is not limited to the skeletal system but can also affect the cardiovascular, gastrointestinal, and metabolic systems.^2^ Furthermore, research has indicated that individuals with AS face a higher likelihood of experiencing metabolic syndrome and cardiovascular issues.^3^

Obesity, characterized by excessive body fat accumulation, has become a major global health concern. It is associated with increased mortality and a higher risk of insulin resistance, metabolic syndrome, diabetes, hypertension, dyslipidemia, and cardiovascular diseases.^4^^,^^5^ Body mass index (BMI) is commonly used to assess overall obesity, while waist circumference (WC) and waist-to-height ratio (WHtR) are indicators of central obesity. Notably, WHtR has been shown to be a more reliable predictor of cardiovascular risk, metabolic syndrome, and insulin resistance compared to BMI, as it reflects central adiposity rather than overall body weight.^6^^,^^7^

Non-alcoholic fatty liver disease (NAFLD) is a common metabolic disorder worldwide, strongly associated with obesity, insulin resistance, and chronic inflammation.^8^ In patients with AS, chronic inflammation and the effects of long-term treatment may further contribute to the risk of NAFLD. However, the prevalence and determinants of NAFLD in this population remain unclear.

Anthropometric parameters such as BMI, WC, and WHtR have been identified as important indicators for evaluating hepatic steatosis.^9^ Additionally, these measures are useful for assessing metabolic complications in AS patients.^10^

There is limited research on the assessment of obesity and NAFLD using ultrasonography (US) in patients with AS. Given the established relationship between NAFLD, metabolic disturbances, and chronic inflammation, it is essential to explore its prevalence in this population. This study aims to evaluate the prevalence of hepatic steatosis in AS patients and investigate its relationship with anthropometric measurements, including BMI, WC, and WHtR, as well as biochemical markers. Understanding these associations is crucial for better management of NAFLD, metabolic risk factors, and potential complications in AS.

## Methods

This was an observational study combining prospectively collected anthropometric data with retrospectively retrieved laboratory and ultrasound records. The study was conducted at the Rheumatology Outpatient Clinic of Antalya Training and Research Hospital. Patients with a diagnosis of AS who were under follow-up between April 2022 and April 2024 and had available abdominal ultrasound data in the hospital records were included. This study was approved by the Ethics Committee of Antalya Training and Research Hospital (Approval No: 6-9, Date: 17-03-2022). Written informed constent was obtained from all participants prior the collection of anthropometric measurements. All procedures were performed in accordance with the Declaration of Helsinki.

Laboratory findings (e.g., liver function tests and lipid parameters) and abdominal ultrasound results were obtained from the hospital’s standardized electronic medical system. Anthropometric measurements (height, weight, and WC) were prospectively recorded by the investigators during routine outpatient visits. Based on these measurements, BMI and WHtR were calculated for each participant.

To minimize temporal bias, efforts were made to ensure that ultrasound and anthropometric assessments were performed within a 3-month interval.

### Patient Selection

This study enrolled 170 individuals aged 18 or older who had been diagnosed with AS based on the modified New York criteria (1984) and were being followed at the Rheumatology Outpatient Clinic of Antalya Education and Research Hospital.

Individuals with a history of consistent alcohol intake (≥40 g per week), chronic liver disorders, type 2 diabetes mellitus, or previously diagnosed hypertension were not included in the study. Additionally, patients diagnosed with metabolic storage diseases or inflammatory bowel disease, as well as those with positive hepatitis markers hepatitis B surface antigen (HBsAg), hepatitis C virus antigen (HCV Ag)(, were excluded. All included patients were physically inactive, with no history of regular exercise. The age, disease duration, and medical treatments of the included patients were recorded. The medical treatments included tumor necrosis factor inhibitors (TNFi) such as adalimumab, infliximab, etanercept, certolizumab, and golimumab.

### Anthropometric Measurements

The height and weight of the patients were measured to calculate the BMI. Body weight (to the nearest 0.1 kg) and height (to the nearest 0.1 cm) were measured, and BMI was calculated by dividing weight in kilograms by the square of height in meters (weight [kg]/height [m]²). Additionally, WC was measured at the midpoint between the lowest rib and the iliac crest after normal expiration. The WHtR was calculated by dividing the WC by the height (WC [cm]/height [cm]).

Body mass index was classified according to the World Health Organization (WHO) criteria as follows: normal: 18.5-24.9 kg/m², overweight: 25.0-29.9 kg/m², obese: ≥30.0 kg/m².^11,12^ The WHtR classification was performed as follows: low risk (healthy): WHtR < 0.5; increased risk (overweight): WHtR 0.5-0.6; high risk (obesity): WHtR > 0.6. These cutoff values are based on previous studies linking WHtR to cardiovascular and metabolic risks.^13^

### Laboratory and Imaging Studies

Laboratory test results were retrospectively obtained from patient records. The analyzed parameters included aspartate aminotransferase (AST), alanine aminotransferase (ALT), high-density lipoprotein (HDL), triglycerides (TGs), erythrocyte sedimentation rate (ESR), and C-reactive protein (CRP).

The presence and severity of hepatic steatosis were retrospectively recorded from ultrasound reports. All abdominal US examinations were performed using a V8 ultrasound system (Samsung Medison, Seoul, Korea) equipped with a Samsung CA1-7S curved-array transducer operating at a frequency range of 1.0 MHz to 7.0 MHz. Hepatic steatosis was assessed using a visual grading system (Grade 0-3) based on grayscale US images, defined as follows:

Grade 0: Normal liver echogenicity with no signs of steatosis.
Grade 1: Mildly increased liver echogenicity with normal visualization of the portal vein wall and diaphragm.
Grade 2: Moderately increased liver echogenicity with reduced visibility of the portal vein wall and diaphragm.
Grade 3: Severely increased liver echogenicity with minimal to no visualization of the portal vein wall and diaphragm.^14^

### Statistical Methods

The statistical analyses of the data obtained in the study were performed using the SASsoftware, version 9.4 (SAS Institute Inc.; Cary, NC, USA). Descriptive statistics for the quantitative variables determined by measurement were presented as mean and standard deviation, while descriptive statistics for the qualitative variables determined by count were presented as number and percentage. Chi-square analysis was conducted to reveal the relationship between qualitative variables. The suitability of the variables used in the study for normal distribution was first tested using the Kolmogorov-Smirnov test. In addition, skewness values were also examined for this purpose. As a result of the tests conducted, since the skewness coefficients for all variables were not between +3 and −3, it was concluded that the data did not show a normal distribution, and non-parametric tests were utilized in the statistical analysis. The Mann–Whitney *U*-test was applied for the comparisons between 2 independent groups, and the Kruskal–Wallis H test was applied for the comparisons between 3 or more independent groups. The data were analyzed for sensitivity and specificity values obtained from receiver operating characteristic (ROC) curves. To create optimal threshold values for the index tests, ROC curves were generated, and the areas under the curve (AUCs) were determined. The ROC curve is a graph that plots the true-positive rates (sensitivity) against the false-positive rates (1-specificity) for different threshold values of the index tests. The closer the curve is to the top left corner of the graph, the higher the AUC and the greater the accuracy of the diagnostic test. Several ROC analyses were performed to assess the accuracy of each index test. In this study, the cutoff value was calculated from the areas under the ROC curves (AUC) of WC samples. The threshold value was selected using the Youden index (= Sensitivity + Specificity − 1). In the entire study, a significance level of .05 was accepted.^15^

## Results

### General Characteristics

A total of 170 patients with AS were included in the study. Of these, 67% (n = 114) were male and 33% (n = 56) were female. The mean age of the patients was 41 years, and the mean disease duration was 7.5 years.

Among the 170 AS patients, 57% (n = 97) were diagnosed with NAFLD, while 43% (n = 73) had no hepatic steatosis. Of the 97 patients with NAFLD, 42% (n = 41) had Grade 1, 46% (n = 44) had Grade 2, and 12% (n = 12) had Grade 3 hepatic steatosis. Additionally, 68% of AS patients were receiving TNFi treatment.

The mean BMI was 29 kg/m², the mean WC was 99 cm, and the mean WHtR was 0.6. Furthermore, among the 97 patients with NAFLD, 66% had an AST/ALT ratio of <1 (Table 1).

### Gender-Based Comparison of Clinical Characteristics

The prevalence of NAFLD was 55% in females and 58% in males. No significant difference was observed between males and females regarding the presence of NAFLD and its severity (* P *= .753, *P *= .929).

There was no significant difference in TNFi use between genders (* P *= .511). The mean age of female patients was significantly higher than that of male patients (44 vs. 40 years, *P *= .011).

Male patients had a significantly larger WC than females (101 vs. 94 cm, *P *= .010). In contrast, BMI and WHtR values did not differ significantly between genders (* P *= .773, *P *= .770).

Aspartate aminotransferase and ALT levels were significantly higher in males compared to females (* P *= .027 and *P *< .001, respectively). Furthermore, the AST/ALT ratio was <1 in males and >1 in females, and this difference was statistically significant (* P *= .0004).

Female patients had higher HDL levels compared to males (57 vs. 45 mg/dL,*P* < .001), while TG levels were lower in females (129 vs. 177 mg/dL, *P *= .006). However, total cholesterol (TC) levels did not differ significantly between genders (* P *= .460).

There was also no significant difference in CRP and ESR levels between males and females (* P *= .395 and *P *= .083, respectively).

The gender-based characteristics of the patients are presented in Table 2.

### Comparison of Patients with and Without Non-Alcoholic Fatty Liver Disease

The mean age of patients with NAFLD was significantly higher compared to those without NAFLD (45 vs. 37 years, *P *< .001).

Among NAFLD patients, 69% were using TNFi, compared to 66% in the non-NAFLD group, with no significant difference between the groups (* P *= .647). There was no statistically significant association between TNFi use and NAFLD.

There was no significant difference in ESR levels between the groups (*P* = .082); however, CRP levels were significantly higher in the NAFLD group (* P *< .001).

Body mass index, WHtR, and WC values were significantly higher in patients with NAFLD (* P *< .001).

There was no significant difference in AST levels (* P *= .282), whereas ALT levels were significantly higher in the NAFLD group (* P *< .001).

High-density lipoprotein levels were significantly lower in the NAFLD group (* P *= .039), whereas TG and TC levels were significantly higher (* P *< .001 and *P *= .0283, respectively).

The AST/ALT ratio was significantly lower in the NAFLD group (<1), and this difference was statistically significant (* P *< .001).

The comparison of patients with and without NAFLD is presented in Table 3.

### Relationship Between Non-Alcoholic Fatty Liver Disease Severity and Patient Characteristics

There was no statistically significant association between TNFi use and NAFLD severity (* P *> .05).

Although ESR did not show a significant difference across NAFLD grades *( P *= .1448), CRP levels increased significantly with hepatic steatosis severity (* P *= .0006). C-reactive protein levels were the highest in patients with Grade 3 NAFLD.

As the severity of NAFLD increased, mean age also increased significantly (* P *< .0001), with the highest mean age observed in patients with Grade 2 NAFLD.

Similarly, BMI, WC, and WHtR increased significantly with NAFLD severity (* P *< .001).

This finding supports the association between hepatic steatosis, obesity, and central adiposity.

Alanine aminotransferase, TG, and TC levels significantly increased with worsening NAFLD severity (* P *= .0013, *P *= .0003, *P *= .0119, respectively).

There was no statistically significant difference in AST levels among NAFLD grades (* P *= .0795).

Similarly, HDL levels did not show a significant change across NAFLD severity (* P *= .1199).

The relationship between NAFLD severity and patient characteristics is presented in Table 4.

### Association of Body Mass Index and Waist-to-Height Ratio with Non-Alcoholic Fatty Liver Disease and Metabolic Parameters

In Table 5, the distribution of NAFLD and metabolic parameters according to BMI and WHtR groups was analyzed. According to BMI classification, the prevalence of NAFLD was 15% in normal-weight individuals, 52% in overweight individuals, and 85% in obese individuals. Based on WHtR classification, NAFLD was detected in 13% of individuals in the low-risk group, 48% in the increased-risk group, and 90% in the high-risk group. These findings indicate that as BMI and WHtR increase, the prevalence of NAFLD rises significantly (* P *< .0001).

C-reactive protein levels also increased significantly with rising BMI and WHtR values (* P *< .0001), supporting the association between inflammation and NAFLD. A strong correlation was observed between obesity markers and abdominal fat accumulation. As BMI and WHtR values increased, WC expanded (* P *< .0001), and ALT levels also significantly increased in terms of liver enzyme parameters (* P *< .0001).

Regarding metabolic parameters, TG and TC levels significantly increased with higher BMI and WHtR values (* P *< .0001 and *P *= .0011, respectively). However, HDL levels did not show a significant difference between BMI and WHtR groups (* P *= .3515). The AST/ALT ratio decreased as BMI and WHtR values increased and fell below 1 in the presence of obesity (* P *< .0001). This finding supports the impact of increasing obesity on liver function and the biochemical changes associated with NAFLD.

### Diagnostic Value of Waist Circumference in Predicting Hepatic Steatosis

An ROC analysis was performed for male and female patients to evaluate the predictive ability of WC for hepatic steatosis.

For male patients, the AUC was calculated as 0.842 (95% CI: 0.771-0.912), with an optimal cutoff value of 100 cm. Patients with a WC above this threshold were found to have a higher risk of hepatic steatosis. The sensitivity of WC was 83.3%, specificity was 72.7%, positive predictive value (PPV) was 69.0%, and negative predictive value (NPV) was 85.7%.

**Figure 1.**
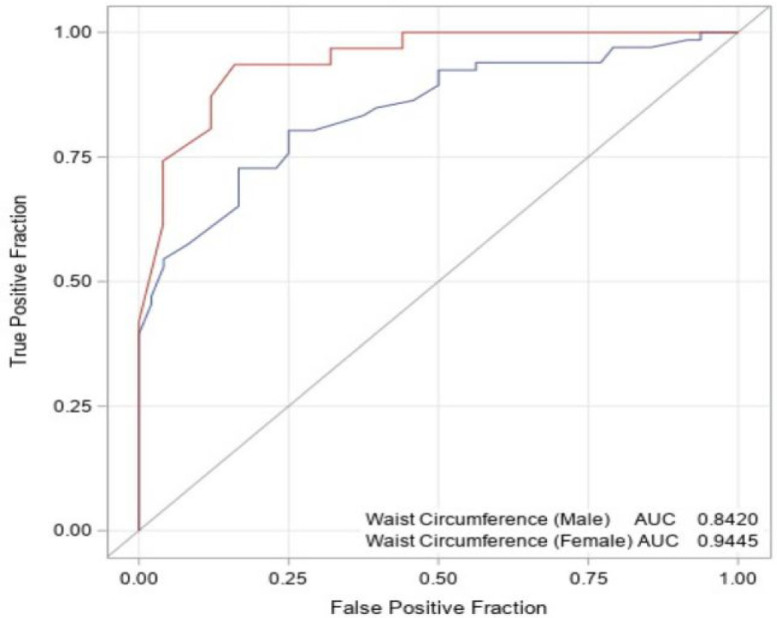
Receiver operating characteristic curve analysis of waist circumference for non-alcoholic fatty liver disease. Red = Female, Blue = Male.

For female patients, ROC analysis yielded an AUC of 0.9445 (95% CI: 0.8893-0.9998), suggesting that WC may serve as a stronger predictor of hepatic steatosis in females. The optimal cutoff value for females was 90 cm, with a sensitivity of 84.0%, specificity of 93.5%, PPV of 91.3%, and NPV of 87.9% (Table 6, Figure 1).

These findings indicate that WC is associated with hepatic steatosis in both male and female patients. However, the determined cutoff values differ between sexes, and the higher specificity in females suggests that WC may be a more reliable predictor in this group.

## Discussion

This study aimed to investigate the relationship between total and central obesity and NAFLD in patients with AS. Additionally, the association of NAFLD and obesity was examined with treatment modalities, lipid profiles, inflammatory markers, and liver function tests.

Non-alcoholic fatty liver disease is a growing global health concern, particularly linked to metabolic disorders and liver diseases. The prevalence of NAFLD in the general population has increased from 25% in 1990-2006 to 38% in 2016-2019. The highest prevalence rates have been reported in Latin America and the Middle East.^8^ Hepatic steatosis has been detected in 50%-75% of diabetic patients,^16^^,^^17^ while approximately 65% of patients with psoriatic arthritis (PsA), another type of spondyloarthritis, have been found to have NAFLD.^18^ In this study, the prevalence of NAFLD in AS patients was 57%, suggesting that NAFLD may be common in AS patients, similar to reports in PsA and diabetes populations.

In individuals with AS, inflammatory markers such as ESR and CRP play a key role in evaluating disease activity and are frequently incorporated into composite disease activity indices.^19^^,^^20^

A study investigating the relationship between obesity and disease activity in AS patients found that obese individuals had higher CRP levels, increased disease activity, and more pronounced radiographic damage.^21^ Similarly, this study demonstrated a significant increase in CRP levels with higher BMI and WHtR scores in AS patients. Additionally, as the severity of NAFLD increased, CRP levels also showed a marked elevation. However, no significant changes were observed in ESR levels. These observations raise the possibility that CRP levels might be affected not only by inflammatory activity but also by factors such as obesity and hepatic steatosis in AS patients.

Studies investigating the relationship between TNF inhibitor (TNFi) use and NAFLD have yielded conflicting results. For instance, a study on patients with inflammatory bowel disease (IBD) reported a higher prevalence of NAFLD among those who were not receiving TNFi therapy.^22^ In contrast, another study found an increased prevalence of NAFLD in IBD patients undergoing TNFi treatment.^23^ In this study, no significant association was observed between TNFi use and hepatic steatosis in AS patients. These divergent findings highlight the need for further comprehensive research to clarify the potential effects of TNFi therapy on NAFLD development.

Non-alcoholic fatty liver disease has been strongly associated with obesity and an increased risk of cardiovascular diseases.^24^^,^^25^ Inflammatory rheumatic diseases such as AS and rheumatoid arthritis have also been linked to higher metabolic syndrome prevalence, further contributing to cardiovascular risk.^3^ A study evaluating NAFLD in living liver donors demonstrated a direct relationship between NAFLD (confirmed by biopsy) and BMI. No NAFLD was detected in individuals with BMI < 25, whereas the prevalence was 33% in those with BMI 25-28 and 76% in those with BMI > 28.^9^ Similarly, this study showed a significant association between obesity, WC, and NAFLD in AS patients. Both BMI and WHtR were higher in the NAFLD group, and WC was also increased. When BMI and WHtR categories were analyzed, the prevalence and severity of NAFLD were highest in the obese group. These findings suggest a potential link between obesity-related parameters and NAFLD in AS patients, warranting further studies to assess their clinical relevance, particularly in the context of cardiovascular risk.

Several studies have highlighted WC as a practical and reliable indicator for predicting NAFLD, independent of BMI and other metabolic markers. A large cohort study demonstrated that individuals with increasing WC trajectories over time had a significantly higher risk of developing NAFLD, even in the absence of abdominal obesity.^26^ Furthermore, research in obese adolescents showed that higher WC quartiles were strongly correlated with NAFLD prevalence, reinforcing the role of WC as an early screening tool.^27^

The WHO defines central obesity as WC ≥ 94 cm in men and ≥ 80 cm in women.^28^ In this study, the prevalence of NAFLD in AS patients was found to be 57%, indicating that NAFLD is not uncommon in this patient population. Furthermore, the ROC analysis identified a WC cutoff value of 100 cm in men and 90 cm in women as optimal for predicting NAFLD in AS patients. Given these findings, WC measurement appears to be a practical and cost-effective tool for assessing NAFLD risk in AS patients. Moreover, WC measurement could serve as an early screening parameter, facilitating timely interventions and better metabolic risk management in clinical practice.

Non-alcoholic fatty liver disease has been strongly linked to alterations in lipid metabolism and liver enzymes. Elevated levels of ALT and TGs have been widely recognized as markers of NAFLD, reflecting increased hepatic fat accumulation and metabolic dysfunction.^29^ Additionally, studies have reported an association between NAFLD and TC, further supporting its role in dyslipidemia.^30^

In this study, patients with AS who had NAFLD exhibited significantly elevated levels of ALT, triglycerides, and TC compared to those without NAFLD. Additionally, HDL levels were notably lower among individuals with NAFLD (*P* = .039), indicating a possible link between NAFLD and decreased HDL concentrations.

However, when the severity of NAFLD was assessed, no significant association was found between HDL levels and NAFLD grade, which aligns with studies indicating that NAFLD primarily influences triglyceride and TC metabolism rather than HDL levels.^31^

Interestingly, AST levels did not significantly differ between patients with and without NAFLD in this study. While ALT is more specific for hepatic fat accumulation, AST may be less sensitive in detecting early-stage NAFLD.^30^ The AST/ALT ratio has also been reported to be below 1 in 65%-90% of patients with NAFLD, indicating its potential diagnostic utility.^29^^,^^31^ In this study, 66% of AS patients with NAFLD exhibited an AST/ALT ratio of <1, which is consistent with the literature. These findings suggest that elevated ALT and TG levels, a reduced HDL concentration, and an AST/ALT ratio of <1 may serve as useful biochemical indicators of NAFLD in AS patients. These parameters highlight the importance of metabolic evaluation in this population.

### Limitations

First, although the study had a prospective component regarding anthropometric measurements, the retrospective nature of laboratory and ultrasound data limits the ability to establish causal relationships between variables. Future prospective studies with complete data collection are warranted to validate these findings.

Second, ultrasonography (US) was used for the diagnosis of NAFLD because of its non-invasive nature and wide availability in clinical practice; however, it lacks the diagnostic accuracy of liver biopsy, which remains the gold standard.

Third, while the study assessed the use of TNF inhibitors, other potential contributors to NAFLD, such as genetic predisposition, dietary habits, and physical activity levels, were not evaluated.

Finally, this was a single-center study, which may limit the generalizability of the results.

To validate these results across different populations, further large-scale and multicenter research efforts are warranted.

Despite these limitations, this study provides valuable insights into the relationship between obesity indices and NAFLD in patients with AS and underscores the need for further investigation in this area.

This study demonstrated that NAFLD is common in AS patients, with a prevalence of 57%. Increased BMI, WHtR, and WC were significantly associated with NAFLD, highlighting the importance of obesity and central adiposity in this population. The ROC analysis identified WC cutoff values of 100 cm in men and 90 cm in women as optimal for predicting NAFLD, suggesting that WC measurement could serve as a simple and cost-effective screening tool in clinical practice.

Additionally, higher ALT, TG, and TC levels, along with lower HDL levels, were significantly associated with NAFLD in AS patients. The AST/ALT ratio was <1 in 66% of patients with NAFLD, which aligns with previous literature and may serve as an additional biochemical indicator. However, no significant association was found between NAFLD and TNFi use, suggesting that other metabolic and inflammatory mechanisms play a more dominant role in hepatic steatosis in AS.

Given the increasing recognition of metabolic complications in AS, early identification of NAFLD using simple anthropometric and biochemical markers may help guide clinical monitoring and preventive strategies. Future longitudinal studies are needed to better understand the long-term implications of NAFLD in AS and its potential impact on disease progression and cardiovascular risk.

## Figures and Tables

**Table 1.** Demographic and Clinical Characteristics of Patients (N = 170)

**Variable**	**Mean (SD)**	**Median (Range)**
Sex, n (%)		
Male	114 (67.1)	
Female	56 (32.9)	
Hepatic steatosis, n (%)		
No fatty liver	73 (42.9)	
G1	41 (24.1)	
G2	44 (25.9)	
G3	12 (7.1)	
Hepatic steatosis, n (%)		
No fatty liver	73 (42.9)	
NAFLD	97 (57.1)	
TNFi use, n (%)		
Yes	115 (67.6)	
No	55 (32.4)	
ESR (mm/h)	11.9 (8.40)	11.0 (1.0-63.0)
CRP (mg/L)	9.9 (9.25)	7.0 (1.0-4.0)
Age (years)	41.4 (10.12)	41.0 (20.0-66.0)
BMI (kg/m²)	28.8 (5.61)	28.1 (17.7-48.0)
WC (cm)	98.7 (15.61)	98.0 (63.0-137.0)
WHtR	0.6 (0.15)	0.6 (0.4-0.9)
Disease duration (years)	7.5 (5.40)	7.0 (1.0-26.0)
AST (U/L)	22.7 (13.06)	20.0 (11.0-134.0)
ALT (U/L)	25.3 (17.64)	20.0 (6.0-129.0)
HDL (mg/dL)	48.9 (12.87)	48.0 (22.0-105.0)
TG (mg/dL)	161.2 (151.43)	131.5 (39.0-1622.0)
TC (mg/dL)	211.0 (43.36)	195.5 (130.0-407.0)
AST/ALT ratio	1.0 (0.36)	1.0 (0.4-2.3)

ALT, alanine aminotransferase; AST, aspartate aminotransferase; BMI, body mass index; CRP, C-reactive protein; ESR, erythrocyte sedimentation rate; G, grade; HDL, high-density lipoprotein; NAFLD, non-alcoholic fatty liver disease; TC, total cholesterol; TG, triglycerides; TNFi, tumor necrosis factor inhibitor; WC, waist circumference; WHtR, waist-to-height ratio.

**Table 2.** Clinical and Laboratory Characteristics by Gender

**Variable**	**Male (N = 114)**	**Female (N = 56)**	***P***
Hepatic steatosis, n (%)			.929
No fatty liver	48 (42.1)	25 (44.6)	
G1	27 (23.7)	14 (25.0)	
G2	30 (26.3)	14 (25.0)	
G3	9 (7.9)	3 (5.4)	
Hepatic steatosis, n (%)			.753
No fatty liver	48 (42.1)	25 (44.6)	
NAFLD	66 (57.9)	31 (55.4)	
TNFi use, n (%)			.511
Yes	79 (69.3)	36 (64.3)	
No	35 (30.7)	20 (35.7)	
Age (years)			.011
Mean (SD)	40.0 (9.17)	44.1 (11.42)	
Median (Range)	39.5 (20.0, 64.0)	46.5 (20.0, 66.0)	
BMI (kg/m²)			.773
Mean (SD)	28.5 (4.73)	29.4 (7.07)	
Median (Range)	28.1 (17.7, 42.8)	28.1 (18.4, 48.0)	
WC (cm)			.010
Mean (SD)	101.0 (14.68)	94.0 (16.51)	
Median (Range)	100.0 (72.0, 137.0)	92.5 (63.0, 128.0)	
WHtR			.770
Mean (SD)	0.6 (0.16)	0.6 (0.13)	
Median (Range)	0.6 (0.4, 0.9)	0.6 (0.4, 0.7)	
AST			.027
Mean (SD)	23.9 (14.94)	20.4 (7.56)	
Median (Range)	21.0 (11.0, 134.0)	19.0 (12.0, 56.0)	
ALT			.0004
Mean (SD)	27.8 (19.43)	20.1 (11.82)	
Median (Range)	21.5 (9.0, 129.0)	17.5 (6.0, 71.0)	
HDL			<.001
Mean (SD)	45.1 (9.80)	56.8 (14.80)	
Median (Range)	45.0 (22.0, 67.0)	54.0 (32.0, 105.0)	
TG			.0067
Mean (SD)	177.0 (174.75)	129.0 (78.44)	
Median (Range)	138.0 (42, 1622)	111.5 (39.0, 435.0)	
AST/ALT ratio			.0004
Mean (SD)	0.9 (0.33)	1.1 (0.38)	
Median (Range)	0.9 (0.5, 2.2)	1.0 (0.4, 2.3)	

Statistical methods: Categorical variables were analyzed using the chi-square test. Normality of continuous variables was assessed using the Shapiro–Wilk test, and the Kruskal–Wallis test was applied.

ALT, alanine aminotransferase; AST, aspartate aminotransferase; BMI, body mass index; G, grade; HDL, high-density lipoprotein; NAFLD, non alcoholic fatty liver disease; TC, total cholesterol; TG, triglycerides; TNFi, tumor necrosis factor inhibitor; WC, waist circumference; WHtR, waist-to-height ratio.

**Table 3.** Comparison of Patients with and Without Fatty Liver (Non-Alcoholic Fatty Liver Disease)

**Variable**	**No Fatty Liver (N = 73)**	**NAFLD (N = 97)**	***P***
TNFi use, n (%)			.647
Yes	48 (65.8)	67 (69.1)	
No	25 (34.2)	30 (30.9)	
CRP			.0003
Mean (SD)	7.3 (8.05)	11.8 (9.67)	
Median (range)	3.0 (1.0, 30.0)	9.0 (1.0, 54.0)	
Age			<.0001
Mean (SD)	37.0 (9.48)	44.6 (9.36)	
Median (range)	35.0 (20.0, 64.0)	44.0 (21.0, 66.0)	
BMI			<.0001
Mean (SD)	25.4 (4.15)	31.4 (5.19)	
Median (range)	25.4 (17.7, 40.2)	31.0 (20.3, 48.0)	
WC			<.0001
Mean (SD)	87.5 (11.16)	107.1 (12.98)	
Median (range)	88.0 (63.0, 110.0)	106.0 (77.0, 137.0)	
WHtR			<.0001
Mean (SD)	0.5 (0.13)	0.6 (0.15)	
Median (range)	0.5 (0.4, 1.5)	0.6 (0.4, 1.8)	
ALT			.0002
Mean (SD)	21.8 (15.80)	27.9 (18.55)	
Median (range)	18.0 (6.0, 111.0)	22.0 (9.0, 129.0)	
HDL			.0391
Mean (SD)	50.9 (13.07)	47.5 (12.58)	
Median (range)	51.0 (22.0, 97.0)	46.0 (26.0, 105.0)	
TG			<.0001
Mean (SD)	121.3 (63.56)	191.2 (187.67)	
Median (range)	108.0 (39.0, 360.0)	161.0 (48.0, 1622.0)	
TC			.0283
Mean (SD)	201.1 (36.49)	218.5 (46.69)	
Median (range)	187.0 (130.0, 283.0)	217.0 (138.0, 407.0)	
AST/ALT ratio			<.0001
Mean (SD)	1.1 (0.36)	0.9 (0.33)	
Median (range)	1.1 (0.5, 2.3)	0.8 (0.4, 2.2)	

Statistical methods: Categorical variables were analyzed using the chi-square test. The normality of continuous variables was assessed using the Shapiro–Wilk test, and the Kruskal–Wallis test was applied for non-normally distributed variables.

ALT, alanine aminotransferase; AST, aspartate aminotransferase; BMI, body mass index; CRP, C-reactive protein; HDL, high-density lipoprotein; NAFLD, non-alcoholic fatty liver disease; ; TC, total cholesterol;TG, triglycerides; TNFi, tumor necrosis factor inhibitor; WC, waist circumference; WHtR, waist-to-height ratio.

**Table 4.** Liver Ultrasound Findings and Clinical Parameters

**Variable**	**No Fatty Liver (N = 73)**	**Grade 1 (N = 41)**	**Grade 2 (N = 44)**	**Grade 3 (N = 12)**	***P***
TNFi use, n (%)	48 (65.8)	29 (70.7)	31 (70.5)	7 (58.3)	.818
CRP (mg/L)					.0006
Mean (SD)	7.3 (8.05)	9.5 (7.73)	13.4 (11.32)	13.8 (7.92)	
Median (range)	3.0 (1.0, 30.0)	8.0 (1.0, 31.0)	10.5 (1.0, 54.0)	13.0 (3.0, 28.0)	
Age (years)					<.0001
Mean (SD)	37.0 (9.48)	42.0 (10.20)	47.7 (7.56)	42.6 (9.79)	
Median (range)	35.0 (20.0, 64.0)	41.0 (23.0, 66.0)	48.0 (34.0, 63.0)	43.0 (21.0, 54.0)	
BMI (kg/m²)					<.0001
Mean (SD)	25.4 (4.15)	29.5 (4.64)	32.3 (5.00)	34.5 (5.56)	
Median (range)	25.4 (17.7, 40.2)	28.9 (24.4, 48.0)	31.5 (23.8, 48.0)	34.9 (20.3, 41.1)	
WC (cm)					<.0001
Mean (SD)	87.5 (11.16)	100.5 (10.43)	110.8 (12.12)	115.9 (14.18)	
Median (range)	88.0 (63.0, 110.0)	99.0 (80.0, 123.0)	110.0 (82.0, 137.0)	119.0 (77.0, 128.0)	
WHtR					<.0001
Mean (SD)	0.5 (0.13)	0.6 (0.09)	0.7 (0.09)	0.8 (0.32)	
Median (range)	0.5 (0.4, 1.5)	0.6 (0.5, 1.0)	0.7 (0.5, 0.9)	0.7 (0.4, 1.8)	
ALT (U/L)					.0013
Mean (SD)	21.8 (15.80)	26.2 (18.88)	28.7 (18.04)	31.1 (20.26)	
Median (range)	18.0 (6.0, 111.0)	21.0 (11.0, 129.0)	23.0 (9.0, 108.0)	24.0 (13.0, 82.0)	
HDL (mg/dL)					.1199
Mean (SD)	50.9 (13.07)	46.5 (9.82)	49.1 (14.12)	44.8 (15.17)	
Median (range)	51.0 (22.0, 97.0)	45.0 (30.0, 67.0)	47.0 (28.0, 105.0)	39.5 (26.0, 80.0)	
TG (mg/dL)					.0003
Mean (SD)	121.3 (63.56)	158.9 (97.00)	184.0 (131.46)	327.8 (425.58)	
Median (range)	108.0 (39.0, 360.0)	137.0 (60.0, 653.0)	167.0 (48.0, 840.0)	215.5 (77, 1622)	
TC (mg/dL)					.0119
Mean (SD)	201.1 (36.49)	208.0 (44.20)	220.1 (42.03)	248.3 (60.23)	
Median (range)	187.0 (130.0, 283.0)	193.0 (138.0, 341.0)	222.0 (145, 297)	242.5 (177, 407)	
AST/ALT ratio					<.0001
Mean (SD)	1.1 (0.36)	0.9 (0.33)	0.9 (0.31)	1.0 (0.38)	
Median (range)	1.1 (0.5, 2.3)	0.9 (0.5, 2.2)	0.8 (0.4, 2.1)	0.8 (0.6, 1.8)	

Statistical methods: Categorical variables were analyzed using the chi-square test. The normality of continuous variables was assessed using the Shapiro–Wilk test, and the Kruskal–Wallis test was applied for non-normally distributed variables.

ALT, alanine aminotransferase; AST, aspartate aminotransferase; BMI, body mass index; CRP, C-reactive protein; HDL, high-density lipoprotein; TC, total cholesterol; TG, triglycerides; TNFi, tumor necrosis factor inhibitor; WC, waist circumference; WHtR, waist-to-height ratio.

**Table 5.** Liver Fatty Changes and Biochemical Parameters by Body Mass Index and Waist-to-Height Ratio Categories

**Parameter**	**Normal BMI** (N = 40)	**Overweight BMI** (N = 61)	**Obese BMI** (N = 69)	**Low WHtR Risk** (N = 38)	**Increased WHtR Risk (N = 64)**	**High WHtR Risk** (N = 68)	*P*
NAFLD (**%**)	15.0	52.5	85.5	13.2	48.4	89.7	<.0001
CRP (mean ± SD)	6.5 ± 7.4	9.2 ± 8.7	12.5 ± 10.0	5.3 ± 5.9	8.7 ± 8.6	13.6 ± 10.0	<.0001
Age (years) (mean ± SD)	33.9 ± 9.3	42.4 ± 8.2	44.8 ± 10.0	33.6 ± 8.7	41.3 ± 9.0	45.8 ± 9.2	<.0001
WC (mean ± SD)	81.6 ± 9.7	96.2 ± 10.3	110.7 ± 11.7	79.5 ± 8.1	94.9 ± 7.0	112.9 ± 10.2	<.0001
ALT (mean ± SD)	17.7 ± 9.3	24.0 ± 15.7	30.9 ± 20.9	17.2 ± 9.3	26.7 ± 15.7	28.5 ± 21.4	<.0001
HDL (mean ± SD)	50.8 ± 13.6	49.1 ± 11.6	47.7 ± 13.5	51.3 ± 15.1	49.0 ± 11.2	47.6 ± 13.0	.3515
TG (mean ± SD)	115.9 ± 51.7	142.3 ± 84.2	204.1 ± 214.1	113.4 ± 53.9	142.4 ± 112.1	205.5 ± 201.9	<.0001
TC (mean ± SD)	190.7 ± 28.0	208.6 ± 40.7	225.0 ± 48.1	188.9 ± 28.9	210.6 ± 38.1	223.8 ± 49.7	.0011
AST/ALT ratio (mean ± SD)	1.3 ± 0.4	1.0 ± 0.3	0.8 ± 0.3	1.3 ± 0.4	0.9 ± 0.3	0.9 ± 0.3	<.0001

Statistical methods: Categorical variables were analyzed using the chi-square test. Normality of continuous variables was assessed using the Shapiro–Wilk test, and the Kruskal–Wallis test was applied for non-normally distributed variables.

ALT, alanine aminotransferase; AST, aspartate aminotransferase; BMI, body mass index; CRP, C-reactive protein; HDL, high-density lipoprotein; NAFLD, non-alcoholic fatty liver disease; TC, total cholesterol; TG, triglycerides; WC, waist circumference; WHtR, waist-to-height ratio.

**Table 6.** Receiver Operating Characteristic Curve Analysis of Waist Circumference for Non-Alcoholic Fatty Liver Disease

**Parameter**	**Male**	**Female**
PPV	0.69	0.913
NPV	0.857	0.879
Sensitivity	0.833	0.840
Specificity	0.727	0.935
AUC	0.842	0.9445
WC (cm)	100	90.0
Youden Index	0.561	0.775
Somers’ D	0.684	0.889
Gamma	0.6961	0.9007
Tau-a	0.3364	0.4474
True positive	40	21.0
True negative	48	29.0
False positive	18	2.0
False negative	8	4.0

Youden Index is a measure of test performance, Somers’ D is a measure of association, Gamma denotes Goodman–Kruskal gamma, and Tau-a denotes Kendall’s Tau-a.

AUC, area under the curve; NPV, negative predictive value; PPV, positive predictive value; ROC, receiver operating characteristic; WC, waist circumference.
